# Supplementary material for: Impact of perceived discrimination and coping strategies on well-being and mental health in newly-arrived migrants in Spain
Source: PLoS One. 2023 Dec 22;18(12):e0294295. doi: 10.1371/journal.pone.0294295 (PMC10745147; doi:10.1371/journal.pone.0294295)
Supplement: S1 Table — (DOCX) [file pone.0294295.s001.docx]

**Supporting information**

**S1 Table. Description of codes and categories used in the present study**

| **Code** | **Code description** | **Subcategory** | **Category** |
| --- | --- | --- | --- |
| rage | *anger, revenge, or impotence* |  | **“Negative emotions due to the perceived discrimination”** |
| shame |  |  |  |
| disgust / sadness | *disappointment and feeling bad* |  |  |
| fear | *includes worry, anxiety, and fear to be discriminated against* |  |  |
| guilt |  |  |  |
| feel undervalued or inferior | *feeling worthless or that others do not value you as they should* | Changes in the way they feel about themselves |  |
| feel vulnerable |  |  |  |
| sense of injustice |  |  |  |
| loneliness | *feeling lonely, marginalized, misunderstood, rejected, and abandoned* |  |  |
| humiliation | *includes feeling offended or denigrated* |  |  |
| hypervigilance | *sense of being judged or watched by others before acting* |  |  |
| mixed | *when one or more feelings, emotions, or moods occur together* |  |  |
| westernization or cultural assimilation | *changing their lifestyle and their dress code to look like a local and attract less attention* | Changes to be more accepted by the local community | **“Change in behaviors due to the perceived discrimination”** |
| creating a good image | *creating a good image of oneself and its culture* |  |  |
| not going out |  | Changes in behavior to avoid discriminatory situations |  |
| avoid going alone |  |  |  |
| hypervigilance | *change routes or places where they used to go and be very careful and more concerned over what they say or do* |  |  |
| stop participating in politics |  |  |  |
| self-sufficient | *less caring or solidary with others* |  |  |
| positive adaptation |  | Extreme behavioral changes |  |
| becoming the oppressor | *repeat the same patterns of their bullies* |  |  |
| ignoring / not responding | *ignoring or not responding to discrimination* | Internalized coping strategies - strategies that don't require interaction with others | **“Identified Coping Strategies to face Discrimination-Related Stress”** |
| isolation | *isolating or detaching oneself from the bully or bullies* |  |  |
| self-medication | *self-medication to deal with discrimination* |  |  |
| intellectual activities / leisure / sport | *writing an introspective diary, reading, listening to music, exercising, and cognitive training* |  |  |
| talk with the oppressor | *search for a constructive dialogue with the bully and request to solve common problems* | Externalized coping strategies - social behaviors that implied others |  |
| insult the oppressor | *offend intentionally through words the bully* |  |  |
| denounce | *using social media platforms, recording the discrimination situation in their phones to obtain public attention, calling the police, etc.* |  |  |
| physical fight | *physical fight against the bully* |  |  |
| revenge | *attacking the bully* |  |  |
| seek for comfort | *seek comfort with a meaningful person* |  |  |
| increase solidarity with others | *be more solidary with other peopel* |  |  |
| cry |  |  |  |
| use humor | *as internal thought or engaging with another personal* | Internalized / Externalized coping strategy |  |
| stress | *Stress due to discrimination. Emotional distress response to discrimination.* |  | **“Discrimination-related Stress”** |
| sadness |  | Problems related to mood | **“Mental Health Problems due to Discrimination-related Stress”** |
| depression |  |  |  |
| suicidal thoughts |  |  |  |
| alcohol and marihuana abuse |  | Problems related to substance abuse and addictive behaviors |  |
| misuse of anxiety medications |  |  |  |
| compulsive video gaming |  |  |  |
| anxiety | *anxiety, overthinking, and over worry* | Problems related to anxiety |  |
| nervousness / having nerves | *referring to anxiety* |  |  |
| nail biting |  |  |  |
| eating compulsively |  |  |  |
| sleeping problems |  |  |  |
| psychological enuresis |  |  |  |
| headaches |  | Not classified problems |  |
