# Supplementary material for: Impact of perceived discrimination and coping strategies on well-being and mental health in newly-arrived migrants in Spain
Source: PLoS One. 2023 Dec 22;18(12):e0294295. doi: 10.1371/journal.pone.0294295 (PMC10745147; doi:10.1371/journal.pone.0294295)
Supplement: S2 Table — Note: S = Spanish; E = English; U = Urdu; A = Arab; CH = Chinese. (DOCX) [file pone.0294295.s002.docx]

**Supporting information**

**S2 Table. Sociodemographic characteristics of interviewers**

|  | | | | |  | Domain of languages | | | | | |
| --- | --- | --- | --- | --- | --- | --- | --- | --- | --- | --- | --- |
| Target Language | | Country of Origin | Gender | Occupation/  Credentials | Initials | S | E | U | A | CH |  |
| T1. Urdu | Pakistan | | F | Technician | H | x | x | x |  |  |  |
| T2. Arab | Morocco | | M | Technician and Social Researcher | RHE | x |  |  | x |  |  |
| T3. Chinese | China | | F | Predoctoral student and Social Researcher (MD) | YH | x | x |  |  | x |  |
| T4. Spanish | Spain | | F | Social and Health Researcher (PhD) | BMM | x | x |  |  |  |  |
| T5. Spanish | Spain | | F | Social Researcher (MD) | MCAB | x | x |  |  |  |  |
| T6. Spanish | Mexico | | M | Health Researcher (PhD) | FV | x | x |  |  |  |  |

*Note: S=Spanish; E=English; U=Urdu; A=Arab; CH=Chinese*
