# Supplementary figures and images for: Impact of perceived discrimination and coping strategies on well-being and mental health in newly-arrived migrants in Spain
Source: PLoS One. 2023 Dec 22;18(12):e0294295. doi: 10.1371/journal.pone.0294295 (PMC10745147; doi:10.1371/journal.pone.0294295)

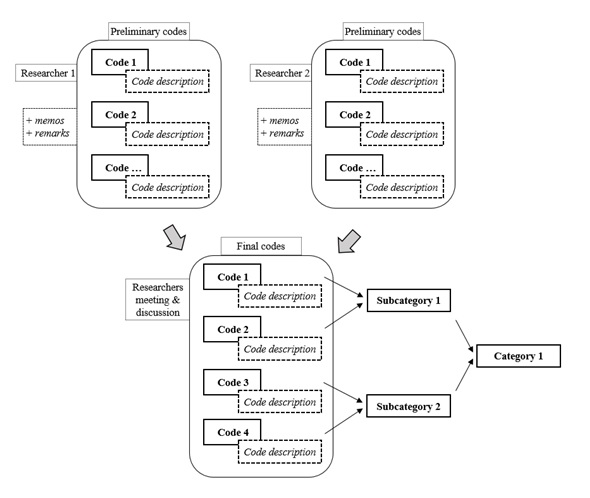

Supplement: S1 Fig — (TIF) [file pone.0294295.s003.tif]

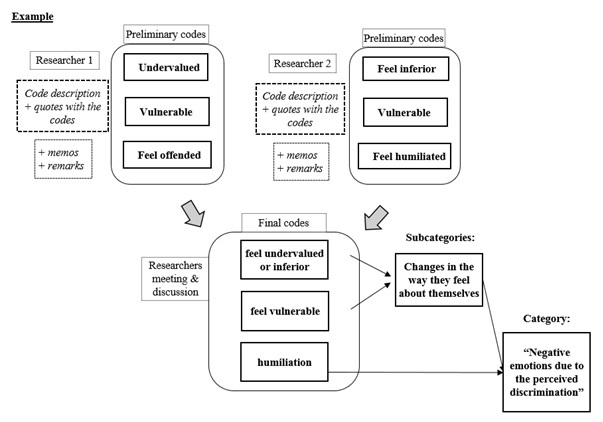

Supplement: S2 Fig — (TIF) [file pone.0294295.s004.tif]
