# Supplementary material for: Impact of perceived discrimination and coping strategies on well-being and mental health in newly-arrived migrants in Spain
Source: PLoS One. 2023 Dec 22;18(12):e0294295. doi: 10.1371/journal.pone.0294295 (PMC10745147; doi:10.1371/journal.pone.0294295)
Supplement: Attachment — Submitted filename: Response to reviewers.docx [file pone.0294295.s005.docx]

Barcelona, 15^th^ September 2023

Dear Editor of PLOS One,

Thank you for the opportunity to improve the manuscript PONE-D-23-16852 entitled “**Impact of Perceived Discrimination and Coping Strategies on Well-being and Mental Health in Newly-Arrived Migrants in Spain”**. We would like to express our sincere gratitude for the suggested corrections. We have addressed all issues raised by the reviewers and revised the Journal’s requirements.

**Author's Reply to Journal requirements**

1. Please ensure that your manuscript meets PLOS ONE's style requirements, including those for file naming.

2. Thank you for stating the financial disclosure. Please address the following queries:

a) Please clarify the sources of funding (financial or material support) for your study. List the grants or organizations that supported your study, including funding received from your institution.

b) State what role the funders took in the study. If the funders had no role in your study, please state: “The funders had no role in study design, data collection and analysis, decision to publish, or preparation of the manuscript.”

**“The funders had no role in study design, data collection and analysis, decision to publish, or preparation of the manuscript.”**

c) If any authors received a salary from any of your funders, please state which authors and which funders.

d) If you did not receive any funding for this study, please state: “The authors received no specific funding for this work.”

Please include your amended statements within your cover letter; we will change the online submission form on your behalf.

3. Thank you for stating the Acknowledgments Section of your manuscript. We note that you have provided funding information that is currently declared in your Funding Statement. However, funding information should not appear in the Acknowledgments section or other areas of your manuscript. We will only publish funding information present in the Funding Statement section of the online submission form.

Please remove any funding-related text from the manuscript and let us know how you would like to update your Funding Statement.

Please include your amended statements within your cover letter; we will change the online submission form on your behalf.

***Reply:*** Thank you for your comments. We have modified our financial disclosure according to the queries in the revised manuscript and removed the funding information from the Acknowledgments section. The updated Funding Statement and Acknowledgments sections are the following:

**Funding Statement**

This work was supported by the Spanish Government with the support of the European Commission under grant [II IN 190517 EN 162 FA 01]. Amanda Lloret-Pineda’s, Blanca Mellor-Marsa’s, Maria del Carmen Alos-Belenguer’s, Yuelu He’s, Rachid El Hafi-Elmokhtari’s, Felipe Villalobo’s, and Ivet Bayes-Marin work was endorsed by the project (II IN 200803 EN 162 FA 01), financed by the Spanish Ministry of Labor, Migrations and Social Security with the support of the European Commission. Aina Gabarrell-Pascuet work was financed by the Secretariat of Universities and Research of the Generalitat de Catalunya and the European Social Fund. Paula Cristóbal-Narváez's work was supported by Sara Borell (CD20/00035) and financed by the Instituto de Salud Carlos III. The funders had no role in study design, data collection and analysis, decision to publish, or preparation of the manuscript.

**Acknowledgements**

The authors would like to express special gratitude to all the participants for their generous contribution, which made this study possible.

4. Please include captions for your Supporting Information files at the end of your manuscript, and update any in-text citations to match accordingly.

***Reply:*** *Thank you, we have added the captions for our Supporting Information at the end of our manuscript.*

5. We note that Figure 1 in your submission contain [map/satellite] images which may be copyrighted. All PLOS content is published under the Creative Commons Attribution License (CC BY 4.0), which means that the manuscript, images, and Supporting Information files will be freely available online, and any third party is permitted to access, download, copy, distribute, and use these materials in any way, even commercially, with proper attribution. For these reasons, we cannot publish previously copyrighted maps or satellite images created using proprietary data, such as Google software (Google Maps, Street View, and Earth). For more information, see our copyright guidelines: <http://journals.plos.org/plosone/s/licenses-and-copyright>.

We require you to either (1) present written permission from the copyright holder to publish these figures specifically under the CC BY 4.0 license, or (2) remove the figures from your submission.

In the figure caption of the copyrighted figure, please include the following text: “Reprinted from [ref] under a CC BY license, with permission from [name of publisher], original copyright [original copyright year].”

Please check copyright information on all replacement figures and update the figure caption with source information. If applicable, please specify in the figure caption text when a figure is similar but not identical to the original image and is therefore for illustrative purposes only.

***Reply:*** Figure 1 was created by the authors using data from the sociodemographic characteristics (i.e., country of origin) of the study sample participants. Therefore, the figure authors are the same as the article authors. The figure was created with the spreadsheet editor “Microsoft Excel” developed by Microsoft for Windows, which has no associated copyright. Below, we attach a link to the official document that explains the printing rights for this type of map and in which you can verify that we can use this type of figure for research purposes. [*https://www.microsoft.com/en-us/maps/bing-maps/product/print-rights/*](https://www.microsoft.com/en-us/maps/bing-maps/product/print-rights/)

6. Please review your reference list to ensure that it is complete and correct. If you have cited papers that have been retracted, please include the rationale for doing so in the manuscript text, or remove these references and replace them with relevant current references. Any changes to the reference list should be mentioned in the rebuttal letter that accompanies your revised manuscript. If you need to cite a retracted article, indicate the article’s retracted status in the References list and also include a citation and full reference for the retraction notice.

***Reply:*** *Thank you, we have carefully revised the reference list.*

**Author's Reply to Reviewer 1**

1) The article necessitates some grammatical revisions, particularly concerning the prohibition of commencing sentences with numerals. Please review the article for a few more minor grammatical errors.

***Reply:*** *Thank you for your comment, we have revised the article for grammatical errors, and we have specifically corrected the sentences commencing with numerals:*

Line 237: **“**Most participants (94 of 102) reported having experienced some type of discrimination since their arrival to the host country.”

Line 232: “Splitting participants by origin (Figure 1), 26 in-depth interviews were conducted with Chinese participants, 21 with Arabic participants from Morocco, 6 with interviewees from other African countries (i.e., Argelia, Cameroon, Mali, Nigeria, Senegal, and Sudan), 40 participants were from Latin America (i.e., Argentina, Brazil, Chile, Colombia, Cuba, Dominican Republic, Honduras, Mexico, Peru, and Venezuela), and 10 from Pakistan and India.”

Line 226: “Considering a non-binary gender approach, we recruited 48 (47.1%) self-identified women, 51 (50.0%) self-identified men, and 3 (2.9%) interviewees reported another gender identity.”

2) Moreover, the term "health problems" should be replaced with "mental health problems" to accurately reflect the focus of the article, which pertains to mental health.
***Reply:*** *Thank you for noticing this; we have corrected the terminology.*

3) The authors have gathered data on variables such as age, gender, education, partnership status, city of residence, and average duration of residency in Spain. Subsequently, did they examine whether any significant associations exist between these variables and their influence on the mental health of the participants?

***Reply:*** *Thank you for your comment. The codes identified arose from the participants’ very diverse narratives. Therefore, each unique code was not represented by a sufficiently significant number of people to associate it with specific variables. Furthermore, as we reported in the Limitations section, since it was a convenience sample, the recruitment of participants focused on vulnerable populations who generally share similar sociodemographic characteristics. Most groups correspond to the same community; for example, the unaccompanied minors are mainly from Morocco, the higher education and socioeconomic level sample is mainly from China, and domestic workers are from Latin America. This means that the only associations we could find were qualitative and mainly linked to the country of origin. The fact that, for example, the sample from Morocco was mainly comprised of young men, could lead us to say that codes associated with this group are associated with young people and the male gender, but this would be a biased statement. Therefore, we believe that due to the type of analysis and sample, we cannot determine these associations. We believe that a future quantitative study could better explore these associations, which would be very interesting to improve understanding of this phenomenon.*

**Author's Reply to Reviewer 2**

Specific Comments:
1) Section 2.4 Data Collection (starting at line 173) should include clear information about the interviewers’ personal characteristics and relationship with participants (COREQ #1-4, 6-8).

***Reply:*** *Thank you for your question. As it is known, many factors influence the chances of success in fieldwork with ethnic minorities, so the proposals to address the recruitment problems fall fundamentally within two areas of action in our study: entry strategies into the community and recruitment and sample diversification strategies. To overcome these barriers and increase the response rate, the researchers were from the same ethnic group - and sometimes the same gender - since it facilitates entry into the community and the relationship with the interviewees. In our study, all researchers received a training course about cross-cultural research and interviewing techniques before starting the recruitment. Participants were interviewed in their native language and by an interviewer from a similar ethnic group (see S2 Table for more information). However, there was no prior personal relationship with any of the participants.*

*We have now specified this in the following paragraph:*

Line 195: Participants were interviewed in their native language and by an interviewer from a similar ethnic group. BMM, MCA-B, YH, REH-E, FV and H conducted the interviews, and they had no prior personal relationship with any of the participants (see Table S2 for more information).

**Table S2**: Sociodemographic characteristics of interviewers

|  | | | |  | Domain of languages | | | | | |
| --- | --- | --- | --- | --- | --- | --- | --- | --- | --- | --- |
| Target Language | Country of Origin | Gender | Occupation/  Credentials | Initials | S | E | U | A | CH |  |
| T1. Urdu | Pakistan | F | Technician | H | x | x | x |  |  |  |
| T2. Arab | Morocco | M | Technician and Social Researcher | REH-E | x |  |  | x |  |  |
| T3. Chinese | China | F | Predoctoral student and Social Researcher (MD) | YH | x | x |  |  | x |  |
| T4. Spanish | Spain | F | Social and Health Researcher (PhD) | BMM | x | x |  |  |  |  |
| T5. Spanish | Spain | F | Social Researcher (MD) | MCA-B | x | x |  |  |  |  |
| T6. Spanish | Mexico | M | Health Researcher (PhD) | FV | x | x |  |  |  |  |

*Note: S=Spanish; E=English; U=Urdu; A=Arab; CH=Chinese*

2) Line 194-195, the authors indicate interviews which were excluded from the analysis. However, if there was the occurrence of refusals or dropouts, the authors did not indicate as such (COREQ #13). Nor, was the presence of non-participants identified (COREQ #15).

***Reply:*** *Thank you for your comment. The dissemination of the study was performed as described in the prior comment, so those individuals that were interested in participating in the study contacted the study research team to be interviewed. As the participation was voluntary and the interview was performed in one session, the study did not have refusals nor drop-outs. Regarding COREQ #15, during the interviews there were only the participant and the interviewer, without any other people present. Following the COREQ guidelines we have added the necessary information to comply with COREQ #15 and #13, respectively:*

Line 179: Due to the COVID-19 pandemic, interviews were administered by phone or using online video-call platforms ensuring data encryption for security and privacy reasons. Five interviews were led in person, as some participants did not know how to use information technologies. During the interviews there were only the participant and the interviewer, to avoid the influence of the presence of non-participants.

Line 201: As the participation was voluntary and the interview was performed in one session, the study did not have refusals or drop-outs.

3) Line 602-603, the last sentence is not necessary “Besides, it would be interesting to transfer the study findings to other stigmatized and oppressed groups in future studies.”
***Reply:*** *Thank you for your suggestion. Following it we have deleted the sentence.*

Major Comments:
4) To better understand the diversity of the study population, the authors should comment on the reason of immigration (e.g. forced migration, economic, etc.). In a related capacity, the manuscript does not comment how this factor may impact the findings of the study. Were there any differential experiences based on this factor? Some explanation is required here.

***Reply:*** *Thank you for your comment. As we mentioned previously, our study aims to guarantee the diversification of the sample, reflecting the heterogeneity of the ethnic migrant groups in terms of discrimination in Spain. In our study, the reasons for migration were heterogeneous and depended on individual characteristics and no patterns were found between participants. For example, in the case of unaccompanied minor male migrants, they reported forced migration by poor living circumstances or voluntary migration, looking for a better future, depending on their personal situation. However, there is no apparent connection with discrimination experiences, and we neither did not directly ask this in the interview because it was not one of our main aims.*

5) In section 3.1 Distribution of Participants (line 221-222), the authors write “94 of 102 participants reported having experienced some type of discrimination since their arrival to the host country”. What were the experiences of the other 8? Was it revealed during the interview they in fact experienced some form of discrimination but did not perceive it as such? How were those interviews considered in the analysis and how might this influence the findings? Some explanation is required here.

***Reply:*** *Thank you for your question, which has greatly enriched the article. For participants who did not report having been discriminated when asked directly, it was revealed that they had experienced some form of discrimination later in their narrative. However, it had not been perceived as such. The interviews were not treated differently, as even though there may not be a perception of discrimination, it does not mean that it does not affect the individual. Furthermore, in all cases, upon reading their narratives, it was evident that they had experienced discrimination.*

Line 237: Most participants (94 of 102) reported having experienced some type of discrimination since their arrival in the host country. Those who did not report having been discriminated against when asked directly, later in their narrative, it was revealed that they had experienced some form of discrimination. However, it had not been perceived as such. This could be attributed to a lack of knowledge of the concept, not being aware of the phenomenon, or due to stigmatization, as some participants reported that for them, it meant having low self-esteem and a debility trait. An example of this experience was described by Participant 18 (P18):

P18: *I've never experienced it [discrimination], but similar things have happened to me, for example, there's people in the street with their phone and they are scared or walk away from the Moroccan [referring to himself], or you make a question to someone, and he/she doesn't answer....* [18 years, from Morocco]
